# Effectiveness of a Pediatric Emergency Medicine Curriculum in a Public Tanzanian Referral Hospital

**DOI:** 10.5811/westjem.2019.10.44534

**Published:** 2019-12-19

**Authors:** Carol C. Chen, Alexander L. Werne, Katharine A. Osborn, Holly Vo, Upendo George, Hendry Sawe, Newton Addo, Andrea T. Cruz

**Affiliations:** * University of California, San Francisco, Section of Pediatric Emergency Medicine, Department of Emergency Medicine, San Francisco, California; † University of California, San Francisco, Department of Pediatrics, San Francisco, California; ‡ University of Utah, Division of Emergency Medicine, Department of Pediatrics, Salt Lake City, Utah; § Muhimbili National Hospital, Department of Emergency Medicine, Dar Es Salaam, Tanzania; ¶ University of California, San Francisco, Department of Medicine, Clinical Pharmacology Program, San Francisco, California; || University of California, San Francisco, Department of Emergency Medicine, San Francisco, California

## Abstract

**Introduction:**

The World Health Organization recently recognized the importance of emergency and trauma care in reducing morbidity and mortality. Training programs are essential to improving emergency care in low-resource settings; however, a paucity of comprehensive curricula focusing specifically on pediatric emergency medicine (PEM) currently exists. The African Federation for Emergency Medicine (AFEM) developed a PEM curriculum that was pilot-tested in a non-randomized, controlled study to evaluate its effectiveness in nurses working in a public Tanzanian referral hospital.

**Methods:**

Fifteen nurses were recruited to participate in a two-and-a-half-day curriculum of lectures, skill sessions, and simulation scenarios covering nine topics; they were matched with controls. Both groups completed pre- and post-training assessments of their knowledge (multiple-choice test), self-efficacy (Likert surveys), and behavior. Changes in behavior were assessed using a binary checklist of critical actions during observations of live pediatric resuscitations.

**Results:**

Participant-rated pre-training self-efficacy and knowledge test scores were similar in both control and intervention groups. However, post-training, self-efficacy ratings in the intervention group increased by a median of 11.5 points (interquartile range [IQR]: 6–16) while unchanged in the control group. Knowledge test scores also increased by a median of three points (IQR: 0–4) in the nurses who received the training while the control group’s results did not differ in the two periods. A total of 1192 pediatric resuscitation cases were observed post-training, with the intervention group demonstrating higher rates of performance of three of 27 critical actions.

**Conclusion:**

This pilot study of the AFEM PEM curriculum for nurses has shown it to be an effective tool in knowledge acquisition and improved self-efficacy of pediatric emergencies. Further evaluation will be needed to assess whether it is currently effective in changing nurse behavior and patient outcomes or whether curricular modifications are needed.

## INTRODUCTION

The lack of emergency care systems has been associated with lower survival rates in adults and children in low- and middle-income countries (LMICs).^1^,^2^ At the 72^nd^ World Health Assembly, delegates recognized the value of emergency and trauma care in reducing morbidity and mortality, and adopted a resolution that would support countries in the development of systems to deliver timely care to critically ill and injured people.^3^ In addition to needs assessments and standards for equipment and processes to support the development of emergency care systems, training for all cadres of health workers was one of the identified interventions in the resolution.

Numerous studies surrounding emergency medicine (EM) curriculum implementation have demonstrated decreased mortality in adult populations^4^–^6^ without a significant increase in the use of resources or economic burden.^5^–^7^ While nearly 95% of the one million traumatic injuries occurring in children worldwide occur in LMICs,^8^ to date, there remains a paucity of open access and comprehensive, pediatric-focused curricula for emergency and trauma care. Furthermore, of the pediatric curricula that have been implemented, most have only evaluated providers’ self-efficacy and knowledge acquisition.^9^–^12^ A rare few have attempted to show changes in provider behavior or patient outcomes.^13^,^14^

In response, the African Federation for Emergency Medicine (AFEM) assembled a working group with expertise in pediatric EM (PEM) from seven American and African academic institutions to develop a comprehensive PEM curriculum for three different tiers of healthcare professionals that would be made freely available. The curriculum development process was based upon a widely accepted model for medical education.^15^ Curriculum topics were based upon a needs assessment conducted in Tanzania two years prior, and learning objectives were determined by expert consensus review using a modified Delphi process.^16^,^17^ As part of the development process, the implementation and evaluation of the curriculum for the first tier of providers, nurses as described here, was conducted as a pilot study at Muhimbili National Hospital (MHN), the national referral hospital for Tanzania (see Figure 1). Specific efforts were made to broaden the curriculum’s evaluation beyond self-efficacy and knowledge acquisition, to include changes in practice behavior.

## METHODS

This study was a non-randomized, controlled pilot study to evaluate the effectiveness of a novel PEM curriculum in nurses (Tier 1 providers) by examining the association between participation in this curriculum and nurses’ self-efficacy, knowledge, and changes in behavior.

### Setting and Study Population

MHN is the national referral hospital for Tanzania and is located in the capital city, Dar es Salaam. It houses an emergency department (ED) that treats approximately 45–50 pediatric patients (under 18 years old) per day.

**Population Health Research Capsule**

What do we already know about this issue?

*Emergency care reduces morbidity and mortality, but countries in low-resource settings may lack dedicated training, especially in pediatric emergency medicine (PEM)*.

What was the research question?

Would Tanzanian nurses who participate in a novel PEM curriculum demonstrate improved self-efficacy, knowledge, and practice during pediatric emergencies?

What was the major finding of the study?

*Trained nurses showed improved self-efficacy and knowledge, but failed to show increased performance of critical actions during live pediatric resuscitations*.

How does this improve population health?

*This PEM pilot has strengthened the curriculum and will be available to train nurses across Africa, to improve the care of critically ill and injured children*.

Nurses were recruited by ED staff at MHN to participate in the training and were matched to control nurses who worked in the same setting based upon their level of experience. All ED Tier 1 nurses or prehospital providers for whom caring for pediatric patients on a daily basis was within their scope of practice were eligible to be enrolled. Any provider not proficient in the English language was excluded from the study. As a retention strategy, a certificate of completion was provided to all participants who attended at least 80% of the training sessions and completed all measurement tools.

The sample-size calculation was based off change in knowledge scores in previously published literature.^7^,^18^ A minimum of 24 nurses (12 intervention and 12 control) was required to detect a 15% change in test scores.

### Control Group

As mentioned, the control group of nurses was recruited from the same group of ED nurses as those in the intervention group, and were matched to participants in the intervention group based upon level of experience. The majority of the nurses in both groups (> 60%) hold a diploma in nursing (three-year program following secondary school or high school), while the remainder possess either a bachelor’s degree or a certificate in nursing. At baseline, all nurses in the ED receive annual training in pediatric emergencies via the American Heart Association’s Pediatric Advanced Life Support course, and a five-day, multidisciplinary course focusing on pediatric resuscitation and trauma management that was developed by local physicians. In addition, they participate in a monthly course entitled “Basic Emergency Nursing Training,” which includes some basic pediatric resuscitation training. Further exposure to pediatric-specific training for the nurses is more sporadic, with up to 25% of weekly continuing nursing education sessions being relevant to pediatrics.

### Intervention

#### Training

Training sessions were conducted over two and a half days and included 30-minute lectures on nine topics, small-group skills stations and simulated scenarios, as well as scheduled intervals for pre-and post-training measurements and frequent breaks (see Appendix 1). The lectures were delivered by two instructors, one international instructor, and one local instructor, and the small-group sessions were facilitated by an additional three international instructors and two local instructors.

#### Outcomes/Measurements

The non-randomized, controlled study design was approved by institutional review boards at both the University of California San Francisco (UCSF) and MHN. Outcome measurements focused on three well-described dimensions to evaluate training programs.^19^ Primary outcomes were 1) participant self-efficacy (as measured by a survey that used five-point Likert scales to rate participants’ confidence with PEM concepts and skills); and 2) participant knowledge acquisition (as measured by written test scores on a 20-question multiple-choice test). Since no surveys for this target audience existed, items were adapted from previously published and validated surveys, with new content development informed by interviews conducted during a needs assessment and review by local experts (EM specialists in low-resource settings).^16^,^20^–^22^ A similar process for developing questionnaires for educational research has been described.^23^

A secondary outcome was change in participant behavior (as measured with a binary checklist of critical actions for pediatric sepsis, respiratory distress, and trauma). Content validity for all tools was obtained through expert review; and a duplicate survey and written test were used for pre-training and post-training evaluations, demonstrating reliability (see Appendices 3 and 4).^24^ Inter-observer reliability for the checklist was not assessed. Each group completed all measurements both pre- and post-training, except for the changes in participant behavior, which was only able to be measured post-training (see “Limitations” section). These data were collected for seven weeks post-training.

#### Statistical Analyses

We used paired *t* tests to compare means between normally distributed groups and Mann-Whitney *U* test to compare data that were not normally distributed. Performance on the checklist of critical actions was compared using chi-squared or Fisher’s exact tests as appropriate. For all comparisons, a two-tailed p-value less than 0.05 was considered statistically significant.

## RESULTS

A total of 15 nurses participated in the training; however, only 11/15 completed all of the pre- and post-training measurements. Fourteen nurses were recruited as control participants; 11/14 completed all measurements. For the survey measurement of self-efficacy, median ratings were similar between the intervention and control groups; however, overall participant post-training ratings were significantly greater than pre-training ratings in the intervention group. Control participants showed no significant difference between their pre-training and post-training ratings (see Tables 1 and 3).

Similarly, for the test of knowledge acquisition, no pre-training difference existed between the intervention and control groups; however, a significant increase in median scores was seen within the intervention group across the two time points, as well as when comparing the intervention group to the control group post-training (see Tables 2 and 3).

A total of 402 live cases of pediatric respiratory distress (121 intervention; 281 control) were observed and measured using the critical actions checklist post-training (Table 4). Only one critical action was observed at a higher proportion in the intervention group (+8.6% (confidence interval [CI], −0.8 – 18.1%): “States that the child is in respiratory distress” (which was intended to serve as a proxy for recognition of an emergency condition – see Appendix 2 for the complete tool). For pediatric trauma, 394 live cases were observed (115 intervention; 279 control) with no statistically significant differences in performance of critical actions. For pediatric sepsis, 396 live cases were observed (117 intervention; 279 control). In two related critical behaviors – “States whether the child is or is not anemic” (eg indicating that the nurse checked for anemia) and “Attempts to place IV or IO (if available)” – the intervention group performed these actions at higher rates with estimated differences of + 6.3% (CI, −0.9 – 13.7%) and 12.6% (CI, 2.1 – 23.0%), respectively.

## DISCUSSION

Emergency care has been proven to save lives, and educational curricula have been shown to be one way to effectively and feasibly support the development and expansion of emergency care services in LMICs.^4^–^7^ Few comprehensive, open-access, pediatric-specific emergency curricula exist despite the high burden of critically ill and injured children in these settings. This study describes the pilot implementation of such a curriculum developed by AFEM to fill this gap, and demonstrates its effectiveness in improving both PEM self-efficacy and knowledge. Once finalized, this curriculum will be made freely available via the Internet to be modified and used to train nurses and prehospital providers across the African continent.

Combined pediatric emergency and critical care fellowships are starting to be implemented on the continent.^25^ At the same time that sub-specialty training for physicians in LMICs is being conducted at referral centers, it is important to recognize that many emergencies take place far from these large centers. To ensure that children receive the care they need, nurses and physicians at sites that are further afield also need training in the recognition and initial management of pediatric emergency conditions, but may not have the financial and logistical ability to commit to two or more years of full-time training.

Our curriculum addresses several of these issues. First, while this pilot training included all components and was held over two and a half days, it is designed in a modular fashion so that participants can view the lectures (available both in PowerPoint and PDF formats to accommodate local bandwidth restrictions) at their convenience. The hands-on, small-group skills sessions and simulated cases can be offered in a brief, one-day training with experienced facilitators. This multimodal format has been shown to be preferred by working emergency care nurses, interns, residents, and physicians in a similar setting.^16^ Secondly, the final AFEM curriculum is designed to target multiple different cadres of healthcare workers through its tiered development. The Tier 1 curriculum piloted in this study is directed toward nurses and pre-hospital providers, recognizing that in most LMICs, the majority of the healthcare workforce is made up of professionals who are not physicians.

This study demonstrated a significant improvement in self-efficacy and knowledge scores of participating nurses. As mentioned, multiple studies of educational curricula have shown similar benefits; however, fewer have demonstrated actual changes in behavior. We attempted to show a change in nursing behaviors with this curriculum. However, an improvement in critical actions during specific pediatric emergencies (respiratory distress, trauma, sepsis), which was expected, was not seen for most actions (Table 4).

There are several potential reasons for this. First, critical actions included stating the existence of certain conditions, such as respiratory distress, as a proxy for recognition of the emergency condition. However, observations were being conducted during actual resuscitations and not in a traditional testing environment, so the fact that a nurse did not verbalize critical action statements may not be a true reflection of his or her recognition of these conditions, but rather a reflection of the lack of utility of such statements during live resuscitations. This hypothesis is supported by the observation that rates of performance of critical actions that required statements were relatively low in both groups, ranging from 6–36% (except for “States that the patient is in septic shock”). However, critical actions that followed recognition of these conditions were performed at relatively higher rates, suggesting that nurses may have been acting upon these emergent conditions, even if they were not stating their recognition of them.

Additionally, this pilot study was conducted at only one site (due to a limitation in funding), so there may have been information transfer among the ED nursing staff, which could have led to an improvement in performance of members of the control group, which makes the lack of significance in the rest of the critical actions difficult to interpret. Since pre-training data was not available to help triangulate the results, it is unclear whether participants were unable to transfer the knowledge acquired into action, or knowledge gained spilled over into the control group. The lack of pre-training data also prohibits us from assessing whether the intervention and control groups performed comparably at baseline. However, a significant difference in baseline is unlikely as there was no difference in their pre-training confidence or knowledge scores. The disproportionately higher number of post-training observations in the control group could be due to an unintentional counting of all nurses who did not undergo the training as controls; however, this could not be confirmed due to lack of identifying information.

Other groups have shown that practice change following educational interventions is often difficult to achieve, and our study supports this notion.^5^ However, given the knowledge acquisition and improved confidence after the course, we believe that this does not suggest that such curricula are not effective, but, if the data are accurate, might not be sufficient, and that another component of training such as direct oversight or on-site mentorship is needed. The value of such presence has been stressed by other researchers.^5^ Future studies are needed to confirm or refute the lack of translation of knowledge into practice, and if confirmed to examine the effect of direct oversight on practice change.

## LIMITATIONS

Our study has several important limitations, including small sample size, possible sample contamination, limited value of specific critical actions as described above, and a failure to confirm inter-observer reliability. In addition, the study design was not randomized due to resource limitations and departmental staffing needs; we were provided with a convenience sample of participant nurses that was matched to a group of control nurses. Our pre-training data collection period for observation of critical actions was eliminated due to delays in obtaining IRB approval, making a comparison across the two time periods impossible. In addition, there was selection bias in recruitment, as many of the nurses in the intervention arm had expressed a particular interest in pediatrics and therefore likely had more experience and motivation. The same questions were used pre- and post-training to assess for knowledge acquisition, which could suggest a positive effect of exposure, however, this is likely limited as the control group did not show a significant increase in scores. Due to funding constraints, this pilot was conducted at only one site, which limits the overall generalizability of the findings.

## FUTURE DIRECTIONS

The results of this pilot study have revealed several important areas needing further investigation. As mentioned above, future studies will focus on determining whether true practice change is occurring from this curriculum, as well as the most important factors contributing to behavior change in nurses and physicians in such settings. Interest in re-examining how this curriculum could alter provider behavior and pediatric mortality, especially with larger sample sizes, has already been expressed by institutions in other countries in Africa.

While this study demonstrated an improvement in confidence and knowledge from this curriculum, the ultimate goal is to develop a course that is effective in creating behavior change that leads to a reduction in pediatric mortality. Once we better understand the findings of this research and can make appropriate adjustments to the course, the goal is to make this course freely available to nurses and prehospital providers to download all of the material and adapt it to their needs and their setting. In addition, three local trainers were trained through this pilot study and the hope is that they will later be able to train future trainers to ensure the local sustainability of the course.

The curriculum for Tier 2 providers (eg, clinical officers, intern physicians) is currently being created and will be piloted at a large medical center in West Africa. The results of this study and subsequent planned studies will be used to modify this curriculum as well. Once these two tiers have been modified and piloted, the curriculum for Tier 3 providers (eg, specialist physicians) will be created and similarly evaluated.

## Supplementary Information









## Figures and Tables

**Table 1:** Overall median self-efficacy rating (scale 11–55), and within cohort p-value comparison of change in self-efficacy ratings pre- and post-training.

Cohort	Self-efficacy assessment	Median rating	Interquartile range	P-value
Control	Pre-	46	41 – 48	0.55
	Post-	45	43 – 49	
Intervention	Pre-	45	39 – 48	0.002
	Post-	54	54 – 55	

**Table 2:** Overall median knowledge score (scale 0–20), and within cohort p-value comparison of change in knowledge scores pre- and post-training.

Cohort	Self-efficacy assessment	Median rating	Interquartile range	P-value
Control	Pre-	14	14 – 17	0.54
	Post-	15	13 – 17	
Intervention	Pre-	16	14 – 17	0.016
	Post-	17	17 – 19	

**Table 3:** P-value comparisons at pre-training and post-training time points between cohorts (intervention vs control).

Time point	Self-Efficacy	Knowledge
Pre-training	p = 0.79	p = 0.53
Post-training	p < 0.001	p = 0.014

**Table 4:** Post-training performance on each critical action (Yes/No) for both intervention and control groups, with associated p-values for comparison across groups.

Respiratory distress (N=402)	Intervention (N=121)			Control (N=281)			Intervention vs. control (%)	P-value
	N/A	No	Yes	N/A	No	Yes		
States: is in respiratory distress	1	90	30	0	235	46	(25.0 vs. 16.4)	0.04
Calls for more resources	27	22	72	111	30	140	(76.6 vs. 82.4)	0.26
Checks respiratory rate	1	109	11	0	264	17	(9.2 vs. 6.0)	0.26
Ensures proper airway alignment	1	103	17	0	246	35	(14.2 vs. 12.5)	0.64
Initiates oxygen therapy	88	8	25	228	8	45	(75.8 vs. 84.9)	0.29
States: whether due to an upper or lower airway condition	1	101	19	0	250	31	(15.8 vs. 11.0)	0.18
Chooses correct-sized mask	94	12	15	241	20	20	(55.6 vs. 50.0)	0.80
Ensures adequate mask-face seal	94	14	13	241	17	23	(48.1 vs. 57.5)	0.47
Assesses chest rise with ventilation	94	11	16	240	21	20	(59.3 vs. 48.8)	0.40
If no chest rise, repositions airway	95	12	14	241	21	19	(53.8 vs. 47.5)	0.62

Trauma (N=394)	Intervention (N=115)			Control (N=279)			Intervention vs. control (%)	P-value
	N/A	No	Yes	N/A	No	Yes		

States: is a trauma patient	1	79	35	0	176	103	(30.7 vs. 36.9)	0.29
Calls for more resources	21	18	76	105	35	139	(80.9 vs. 79.9)	0.85
States: assessment of airway	0	76	39	0	178	101	(33.9 vs. 36.2)	0.67
States: assessment of breathing	1	78	36	2	185	92	(31.6 vs. 33.2)	0.81
States: assessment of circulation	1	79	35	1	192	86	(30.7 vs. 30.9)	0.96
States: patient’s GCS or AVPU	0	98	17	0	234	45	(14.8 vs. 16.1)	0.74
Exposes entire body with modesty	2	76	37	2	201	76	(32.7 vs. 27.4)	0.33
States: need for neck stabilization	0	110	5	0	262	17	(4.3 vs. 6.1)	0.49
Applies splint to extremity	105	1	9	217	8	54	(90 vs. 87.1)	0.80

Septic Shock (N=396)	Intervention (N=117)			Control (N=279)			Intervention vs. control (%)	P-value
	N/A	No	Yes	N/A	No	Yes		

States: child is in septic shock	0	56	61	0	142	137	(52.1 vs. 49.1)	0.66
Calls for more resources	22	20	75	105	33	141	(78.9 vs. 81.0)	0.68
States: if child is malnourished	0	90	27	0	233	46	(23.1 vs. 16.5)	0.12
States: if child is anemic	0	102	15	0	261	18	(12.8 vs. 6.5)	0.04
Attempts intravenous or intraosseous access	0	31	86	0	109	170	(73.5 vs. 60.9)	0.02
Gives correct fluid resuscitation for child without anemia/malnutrition	61	17	39	202	32	45	(69.6 vs. 58.4)	0.19
Gives correct fluid resuscitation for child with malnutrition	76	21	20	210	31	38	(48.8 vs. 55.1)	0.52
States: need blood transfusion for fluid resuscitation if severe anemia	95	17	5	265	12	2	(22.7 vs. 14.3)	0.68

Note: Phrasings in the table are abbreviations; refer to Appendix 2 for original checklist items.

*N/A*, not applicable.

## References

[1] Hsiao M, Morris SK, |Malhotra A (2013). Time-critical mortality conditions in low-income and middle-income countries. Lancet.

[2] Reynolds TA, Sawe H, Rubiano AM, Shin SD, Wallis L, Mock CN, Jamison DT, Gelband H, Horton S (2017). Chapter 13: Strengthening health systems to provide emergency care. Disease Control Priorities: Improving Health and Reducing Poverty.

[3] Director-General (2019). Emergency care systems for universal health coverage: ensuring timely care for the acutely ill and injured.

[4] Wang P, Li NP, Gu YF (2010). Comparison of severe trauma care effect before and after advanced trauma life support training. Chin J Traumatol.

[5] Petroze RT, Byiringiro JC, Ntakiyiruta G (2015). Can focused trauma education initiatives reduce mortality or improve resource utilization in a low-resource setting?. World J Surg.

[6] Arreola-Risa C, Mock C, Herrera-Escamilla AJ (2004). Cost-effectiveness and benefit of alternatives to improve training for prehospital trauma care in Mexico. Prehosp Disaster Med.

[7] Crouse HL, Vaides H, Torres F (2016). Quality and effectiveness of a pediatric triage training program in a Guatemalan public hospital. Pediatr Emerg Care.

[8] Mirkuzie AH, Sisay MM, Bedane MM (2014). Standard basic emergency obstetric and neonatal care training in Addis Ababa; trainees reaction and knowledge acquisition. BMC Med Educ.

[9] Kiragu AW, Dunlop SJ, Wachira BW (2017). Pediatric trauma care in low- and middle-income countries: a brief review of the current state and recommendations for management and a way forward. J Pediatr Intensive Care.

[10] Chang MP, Walters CB, Tsai C, Sampson J (2019). Evaluation of a neonatal resuscitation curriculum in Liberia. Children (Basel).

[11] Fant CD, Schwartz KR, Patel H (2017). Developing and implementing a pediatric emergency care curriculum for providers at district level hospitals in Sub-Saharan Africa: a case study in Kenya. Front Public Health.

[12] Wesson HK, Plant V, Helou M (2017). Piloting a pediatric trauma course in western Jamaica: lessons learned and future directions. J Pediatr Surg.

[13] Chomba E, Carlo WA, Goudar SS (2017). Effects of essential newborn care training on fresh stillbirths and early neonatal deaths by maternal education. Neonatology.

[14] Carlo WA, Goudar SS, Jehan I (2010). Newborn-care training and perinatal mortality in developing countries. N Engl J Med.

[15] Thomas PA, Kern DE, Hughes MT (2016). Curriculum Development for Medical Education.

[16] Vo H, George U, Straube S, Tenner A, Sawe H, Chen C (2018). A needs assessment of pediatric emergency and critical care in Tanzanian providers: a model for curriculum development. Pediatric Academic Society Global Pediatric Research.

[17] Carman A, Daftary R, George U, Sawe H, Chen C (2019). Development of the objectives for a pediatric emergency medicine curriculum for the African Federation of Emergency Medicine. Western Regional Society for Academic Emergency Medicine.

[18] Tchorz KM, Thomas N, Jesudassan S (2007). Teaching trauma care in India: an educational pilot study from Bangalore. J Surg Res.

[19] Kirkpatrick DL, Kirkpatrick JD (1998). Evaluating Training Programs: The Four Levels.

[20] Spaite DW, Karriker KJ, Seng M (2000). Increasing paramedics’ comfort and knowledge about children with special health care needs. Am J Emerg Med.

[21] Dobson JV, Brancati DS, Nagel R (2003). Pediatric resuscitation: evaluation of a clinical curriculum. Med Educ Online.

[22] Shilkofski N, Crichlow A, Rice J (2018). A standardized needs assessment tool to inform the curriculum development process for pediatric resuscitation simulation-based education in resource-limited settings. Front Pediatr.

[23] Artino J, Anthony R, La Rochelle JS (2014). Developing questionnaires for educational research: AMEE guide no. 87. Med Teach.

[24] Sullivan GM (2011). A primer on the validity of assessment instruments. J Grad Med Educ.

[25] Fellowship training program. PECC-Kenya pediatric emergency and critical care web site.

