# Supplementary material for: Effectiveness of a Pediatric Emergency Medicine Curriculum in a Public Tanzanian Referral Hospital
Source: West J Emerg Med. 2019 Dec 19;21(1):134–40. doi: 10.5811/westjem.2019.10.44534 (PMC6948709; doi:10.5811/westjem.2019.10.44534)
Supplement: Supplementary file 1 [file wjem-21-134-s001.docx]

**Appendix 1. Training schedule**

Day 1

| **Time** | **Activity** |
| --- | --- |
| 8:00 am | Introduction   - Review of course schedule and logistics - Obtain consent |
| 8:30 am | Pre-tests   - Self-efficacy survey - Knowledge test |
| 10:00 am | Tea break |
| 11:00 am | General Principles Module I |
| 11:30 am | General Principles Module II |
| 12:00 pm | Skills Practice and Simulated Scenarios focused on General Principles |
| 1:00 pm | Lunch |
| 2:00 pm | Respiratory Module |
| 2:30 pm | Skills Practice and Simulated Scenarios focused on Respiratory |
| 3:30 pm | Break |
| 4:00 pm | Shock Module |
| 4:30 pm | Review with Question and Answer session   - Additional practice as needed |
| 4:30 - 5:30 pm | Adjourn |

Day 2

| **Time** | **Activity** |
| --- | --- |
| 8:00 am | Review of previous day |
| 8:30 am | Neurology – Seizure Module |
| 9:00 am | Skills Practice and Simulated Scenarios focused on Seizure |
| 9:30 am | Neurology – Altered Mental Status Module |
| 10:00 am | Tea break |
| 11:00 am | Hematology – Anemia Module |
| 11:30 am | Hematology – Sickle Cell Module |
| 12:00 pm | Review with Question and Answer session |
| 12:30 pm | Lunch |
| 1:30 pm | Resuscitation – CPR and Teamwork Skills Module |
| 2:00 pm | Resuscitation - Neonatal Resuscitation Module |
| 2:30 pm | Skills Practice and Simulated Scenarios focused on Resuscitation and Teamwork Skills |
| 4:00 pm | Break |
| 4:30 pm | Electrolytes and GI Module |
| 5:00 pm | Review with Question and Answer session  Additional practice as needed |
| 5:00 - 6:00 pm | Adjourn |

Day 3

| **Time** | **Activity** |
| --- | --- |
| 8:00 am | Review of previous day |
| 8:30 am | Trauma – Module 1 |
| 9:00 am | Trauma – Module 2 |
| 9:30 am | Skills Practice and Simulated Scenarios focused on Trauma |
| 10:30 am | Tea break |
| 11:30 am | Review with Question and Answer session |
| 12:30 pm | Lunch and Post-tests   - Self-efficacy survey - Knowledge test |
| 1:30 – 2:00 pm | Adjourn |
