# Supplementary material for: Effectiveness of a Pediatric Emergency Medicine Curriculum in a Public Tanzanian Referral Hospital
Source: West J Emerg Med. 2019 Dec 19;21(1):134–40. doi: 10.5811/westjem.2019.10.44534 (PMC6948709; doi:10.5811/westjem.2019.10.44534)
Supplement: Supplementary file 2 [file wjem-21-134-s002.docx]

**Appendix 2. Critical Actions Checklist for Tier 1 Providers**

Respiratory Distress:

1. States that child is in respiratory distress YES NO
2. Calls for more resources (if available) YES NO
3. Checks respiratory rate YES NO
4. Ensures proper airway alignment by performing head tilt/chin lift,

jaw thrust, or placing a towel beneath the shoulders YES NO

1. Initiates oxygen therapy by any means, if available YES NO
2. States whether the patient is having respiratory distress from an

upper airway condition or a lower airway condition. YES NO

1. If available, performs proper bag-valve-mask ventilation of a patient

with no spontaneous breathing or inadequate breathing, including:

- 1. Chooses the correct-sized mask (covers nose and mouth

without placing pressure on eyes or fitting past the chin) YES NO

- 1. Ensures adequate mask-face seal YES NO
  2. Assesses chest rise with ventilations YES NO
  3. If no chest rise, repositions airway YES NO

1. States indications for transfer if applicable: YES NO
   1. Patient requiring oxygen
   2. Patient is in severe respiratory distress
   3. Patient in respiratory failure

Trauma:

1. States that either the patient is a trauma patient or that the patient

needs a trauma evaluation. YES NO

1. Calls for more resources (if available) YES NO
2. Performs a primary assessment including:
   1. States an assessment of patient’s airway YES NO
   2. States an assessment of patient’s breathing YES NO
   3. States an assessment of patient’s circulation YES NO
   4. States the patient’s Glasgow Coma Score YES NO
   5. Exposes patient’s entire body while maintaining modesty YES NO
3. States whether or not the child needs immobilization of the cervical

spine. YES NO

1. Applies a splint to an extremity with a suspected fracture, if applicable. YES NO
2. States indications for transfer if applicable: YES NO
   1. Any abnormality of the primary survey
   2. Need for advanced testing, imaging or specialist consultation
   3. Bleeding that cannot be controlled with direct pressure
   4. Bleeding requiring placement of a tourniquet

Septic Shock: Circle one

1. States that patient is in septic shock YES NO
2. Calls for more resources (if available) YES NO
3. States whether patient is or is not malnourished YES NO
4. States whether patient is or is not severely anemic YES NO
5. Attempts to place IV or IO (if available) YES NO
6. Initiates appropriate fluid management
   1. For a child in shock without anemia or malnutrition, initiates

IV/IO/NG/OG fluid resuscitation with 10 -20 mL/kg of normal saline

or Lactated Ringer's over 30 minutes YES NO

- 1. For a child in shock with malnutrition, initiates IV/IO/NG/OG

fluid resuscitation with 10 - 15 mL/kg of dextrose-containing fluids

over 1 hour YES NO

- 1. For a child in shock with severe anemia, states that a blood

transfusion should be given if available YES NO

1. States indications for transfer, if applicable YES NO
   1. Severe anemia
   2. Septic shock in a malnourished child
   3. If the patient needs IV antimicrobial therapy
   4. If there is no improvement or worsening after the first fluid bolus
