# Supplementary material for: Effectiveness of a Pediatric Emergency Medicine Curriculum in a Public Tanzanian Referral Hospital
Source: West J Emerg Med. 2019 Dec 19;21(1):134–40. doi: 10.5811/westjem.2019.10.44534 (PMC6948709; doi:10.5811/westjem.2019.10.44534)
Supplement: Supplementary file 3 [file wjem-21-134-s003.docx]

**Appendix 3. Knowledge Assessment Tool for Tier 1 Providers**

1. Why are infants at higher risk for airway obstruction than adults?

A. Infants breathe slower when compared to adults

**B. Infants have a large head, large tongue, and a small airway when compared to adults**

C. Pediatric patients have large jaws and small tongues

D. There are no anatomical differences between children and adult

1. If an infant is in respiratory distress and has nasal congestion due to illness, what interventions may improve his/her breathing?

**A. Suction the infant’s nares**

B. Give acetaminophen or paracetamol

C. Feed the infant before other interventions

D. Place the infant flat on their back

1. According to ETAT (Emergency Triage Assessment and Treatment), what is the next step when a child is found to have an “Emergency” sign?

A. The child should wait his/her turn in the queue.

B. The child’s caregiver will be notified of additional charges.

**C. The patient needs immediate intervention**

D. A provider will be notified only if the child is less than 2 years of age.

1. Which of the following is NOT an emergency sign according to ETAT (Emergency Triage Assessment and Treatment)?

A. Circulation

B. Breathing

**C. Temperature**

D. Dehydration

1. A 3-year-old boy presents to the emergency department with a cough for the past 2 hours. His mother reports that he was playing outside when suddenly she heard him start coughing. He has had no recent fevers or nasal congestion. On exam, you note that his oxygen saturations are 97% on room air, he is breathing 30 times per minute, and he has significant suprasternal retractions. He can speak to you but is distressed. What is your NEXT BEST step?

A. Immediately intubate the child

B. Administer an oral steroid

**C. Assess the child’s airway**

D. Send the child for a chest x-ray

1. Which size mask is correct for bag-mask ventilation? Answer: **A**


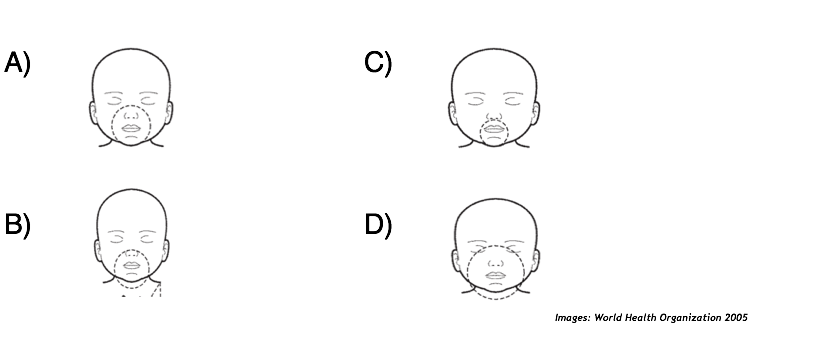


1. An 8-year-old girl is brought to you after a road traffic accident. She is bleeding profusely from an open femur fracture. Her HR is 160, BP is 80/40 and her mental status is altered. You think she is in hemorrhagic shock. Do you transfer her for higher level of care and what is your next BEST action?
2. No; place an IV and give her ceftriaxone
3. No; place an NG tube and give her 20 mL/kg of normal saline or Lactated Ringer’s
4. **Yes; splint the affected leg and apply pressure to stop the bleeding**
5. Yes; leave the affected leg in a position of comfort and do not touch it
6. A 4-year-old girl is brought in with lethargy, fever and fast breathing. Her skin is hot and flushed and her pulse is fast and strong. What is her diagnosis and what do you do for her in the first five minutes?
7. Cold septic shock; place an IV and give adrenaline
8. Hypovolemic shock; give ORS
9. Warm septic shock; place an IV and give steroids
10. **Warm septic shock; place an IV and give 20 mL/kg of normal saline**
11. Which of the following is NOT a sign of hypoglycemia in a child?
12. Seizure
13. Altered mental status
14. Irritability
15. **Frequent urination**
16. A mother brings you her 2-year-old daughter who is having rhythmic jerking of her arms and legs with her eyes rolled backwards. The mother states this has been going on for at least 2 minutes. You suspect she is having a seizure. Your next steps include:
17. Put your hand in the child’s mouth so she does not bite her tongue
18. **Place the patient on her side and monitor her airway and breathing.**
19. Submerge the patient in a cool bath to treat her elevated temperature
20. Wait 10 minutes then give the patient glucose if she is still seizing
21. A 4-year-old boy is brought to you after a high-speed road traffic accident. What is the next best IMMEDIATE step?
22. Begin cleaning out his wounds so they don’t get infected
23. Obtain a thorough history of present illness and past medical history
24. **Assess the patient’s airway, breathing, circulation and disability (level of consciousness) and expose the patient**
25. Perform the head-tilt chin-lift maneuver
26. A 12-year-old girl comes to you after a fall from a tree onto her outstretched right hand. Her arm appears as pictured below. She has a strong radial pulse on the affected side and is easily wiggling her fingers. What is the next best step?


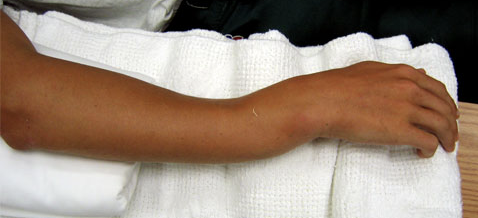


1. **Splint in a position of comfort and transfer to a higher level of care**
2. Immediately attempt a reduction of the affected arm
3. Place an IV and give antibiotics
4. Observation only
5. A 6-year-old boy was hit by a car and is bleeding profusely from a 1 cm x 1 cm area of his scalp. The most effective way to stop the bleeding is to:
6. Wrap his entire head tightly with a bandage
7. Hold a large stack of gauze over the wound
8. Apply a tourniquet
9. **Apply direct pressure on the wound with gloved fingers**
10. A 2-year-old boy is brought in by his mother after boiling water is spilled onto his face and chest. Twenty percent of his body surface area is involved with the burn. The affected skin is blistered and very tender. Does this patient need transfer to a higher level of care?
11. **Yes**
12. No
13. A father brings in his 6-year-old son because he has been more tired than usual and lost consciousness when he stood up this morning. He has not had a fever or diarrhea. You discover that he is breathing fast, has a fast heart rate, and that his conjunctiva and palmar creases are pale, but he is smiling and answering all of your questions appropriately. What is the most likely diagnosis?
14. Seizure
15. Typhoid
16. Cerebral malaria
17. **Severe anemia**
18. You are taking care of a 10-year-old girl with known sickle cell disease. Which of the following is NOT an emergent complication of sickle cell disease?
19. Acute chest syndrome
20. Stroke
21. Vaso-occlusive crises
22. **Dehydration**
23. A 15-month-old boy has been having 16 episodes of watery diarrhea per day for the past 7 days. He is irritable, but alert and reacting appropriately. His eyes are sunken and when you pinch his skin it returns slowly. He is refusing to drink. What is your NEXT BEST step?
24. Since he is alert, he is safe to go home
25. **Place an orogastic tube or nasogastric tube and give oral rehydration solution**
26. Immediately arrange for transfer to a paediatric facility for a peripheral IV and IV fluids
27. Continue to offer him a cup of water to drink

1. A newborn baby is silent, limp and not breathing immediately after she has been delivered. What is the FIRST thing should you do?
2. **Dry the baby thoroughly**
3. Shake the baby vigorously
4. Throw cold water on the baby’s face
5. Hold the baby upside down by its feet
6. An unresponsive 6-year-old boy is brought to you being carried in his father’s arms. His father tells you that the child was running around outside when he collapsed. What is your NEXT BEST step in caring for this patient?
7. Remove all of his clothing to fully expose the patient
8. **Check for a pulse**
9. Obtain IV access
10. Immediately intubate the patient
11. You are transferring a critically ill child to a higher level of care. When handing-off the patient to the next provider who will care for the child you should include all the following in your report EXCEPT:
12. Identify the patient and give a brief background summary about what happened to the patient before you intervened
13. Give your assessment of what you think could be wrong with the child
14. Give your recommendations on what further care the child needs and why you are transferring the patient
15. **All of the above are important components to include in your hand-off report**
