# Supplementary material for: Effectiveness of a Pediatric Emergency Medicine Curriculum in a Public Tanzanian Referral Hospital
Source: West J Emerg Med. 2019 Dec 19;21(1):134–40. doi: 10.5811/westjem.2019.10.44534 (PMC6948709; doi:10.5811/westjem.2019.10.44534)
Supplement: Supplementary file 4 [file wjem-21-134-s004.docx]

**Appendix 4. Survey Tool for Tier 1 Providers**

1) What is your current job title (circle one)?

| Community Health Worker | Pharmacist |  | Pre-hospital provider (e.g. paramedic, ambulance driver) | Nurse | Other (please specify):  ___________ |
| --- | --- | --- | --- | --- | --- |

2) To see if the training is helpful to you and your educational needs, we would like to get your input on the different topics that the course covers. Please be as honest as possible. Your responses will be kept confidential.

*Instructions:* Please rate your current comfort level with performing each of the following medical tasks by circling your response after each statement.

|  | **Not at all comfortable** | **Slightly Comfortable** | **Somewhat comfortable** | **Very comfortable** | **Extremely comfortable** |
| --- | --- | --- | --- | --- | --- |
|  |  |  |  |  |  |
| Identifying the early signs of respiratory failure in a pediatric patient | 1 | 2 | 3 | 4 | 5 |
| Treating a child in hypovolemic shock | 1 | 2 | 3 | 4 | 5 |
| Starting the evaluation and treatment for a seizing child | 1 | 2 | 3 | 4 | 5 |
| Recognizing the signs of hypoglycemia in a child | 1 | 2 | 3 | 4 | 5 |
|  |  |  |  |  |  |
|  | **Not at all comfortable** | **Slightly Comfortable** | **Somewhat comfortable** | **Very comfortable** | **Extremely comfortable** |
|  |  |  |  |  |  |
| Starting fluid management for a malnourished child with signs of shock | 1 | 2 | 3 | 4 | 5 |
| Performing effective bag- valve mask ventilation for an apneic infant in the first minute of life | 1 | 2 | 3 | 4 | 5 |
|  |  |  |  |  |  |
| Performing high quality CPR on a infant and child | 1 | 2 | 3 | 4 | 5 |
|  |  |  |  |  |  |
| Recognizing severe anemia | 1 | 2 | 3 | 4 | 5 |
| Recognizing the severity of a burn and starting appropriate therapy | 1 | 2 | 3 | 4 | 5 |

| Immobilizing the cervical spine for a child after a trauma | 1 | 2 | 3 | 4 | 5 |
| --- | --- | --- | --- | --- | --- |

Please share any thoughts you have that you didn’t feel were addressed by the questions above:
